# The missing link: allostery and catalysis in the anti-viral protein SAMHD1

**DOI:** 10.1042/BST20180348

**Published:** 2019-07-11

**Authors:** Elizabeth R. Morris, Ian A. Taylor

**Affiliations:** Macromolecular Structure Laboratory, The Francis Crick Institute, 1 Midland Road, London NW1 1AT, U.K.

**Keywords:** allosteric regulation, HD domain, HIV, hydrolase, oligomerization, SAMHD1

## Abstract

Vertebrate protein SAMHD1 (sterile-α-motif and HD domain containing protein 1) regulates the cellular dNTP (2′-deoxynucleoside-5′-triphosphate) pool by catalysing the hydrolysis of dNTP into 2′-deoxynucleoside and triphosphate products. As an important regulator of cell proliferation and a key player in dNTP homeostasis, mutations to SAMHD1 are implicated in hypermutated cancers, and germline mutations are associated with Chronic Lymphocytic Leukaemia and the inflammatory disorder Aicardi–Goutières Syndrome. By limiting the supply of dNTPs for viral DNA synthesis, SAMHD1 also restricts the replication of several retroviruses, such as HIV-1, and some DNA viruses in dendritic and myeloid lineage cells and resting T-cells. SAMHD1 activity is regulated throughout the cell cycle, both at the level of protein expression and post-translationally, through phosphorylation. In addition, allosteric regulation further fine-tunes the catalytic activity of SAMHD1, with a nucleotide-activated homotetramer as the catalytically active form of the protein. In cells, GTP and dATP are the likely physiological activators of two adjacent allosteric sites, AL1 (GTP) and AL2 (dATP), that bridge monomer–monomer interfaces to stabilise the protein homotetramer. This review summarises the extensive X-ray crystallographic, biophysical and molecular dynamics experiments that have elucidated important features of allosteric regulation in SAMHD1. We present a comprehensive mechanism detailing the structural and protein dynamics components of the allosteric coupling between nucleotide-induced tetramerization and the catalysis of dNTP hydrolysis by SAMHD1.

## Introduction

**Figure 1.**
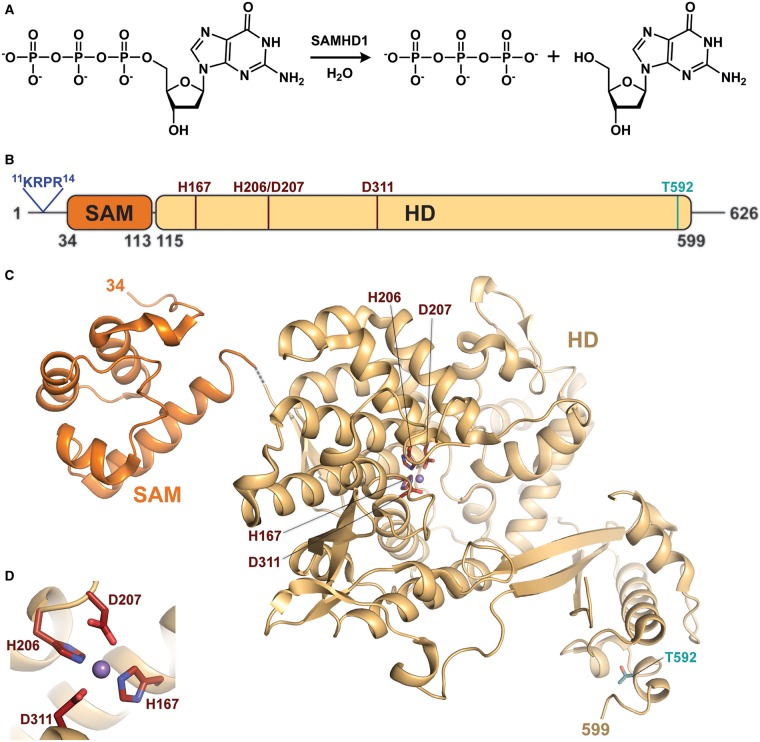
SAMHD1 structure and catalytic activity. (**A**) The dNTP triphosphohydrolysis reaction catalysed by SAMHD1, dGTP is shown as the example substrate. (**B**) Domain organisation of human SAMHD1, showing the nuclear localisation signal in blue, the SAM domain in dark orange, the HD domain in light orange, the HD motif residues in maroon and phosphorylated residue Thr592 in teal. (**C**) Left: NMR structure of the human SAMHD1 SAM domain (PDB code: 2E8O residues 34–113). Right: X-ray crystal structure of WT human SAMHD1 HD domain (PDB code: 4BZC, monomer A, residues 115–599) [85]. The structures of the two domains are connected by a short, dotted and grey line. The HD motif-co-ordinated manganese ion is shown as a purple sphere, and the HD motif residues are shown as maroon sticks. (**D**) A close-up view of the HD motif-co-ordinated manganese ion in the catalytic site. PyMOL was used to prepare the structural figures.

Sterile-α-motif and HD domain containing protein 1 (SAMHD1) is a vertebrate, cellular protein that catalyses the hydrolysis of 2′-deoxynucleoside-5′-triphosphate (dNTP) into 2′-deoxynucleoside and triphosphate (Figure 1A) [1–6]. The replication of some DNA viruses and several retroviruses, such as HIV-1, is restricted by the catalytic activity of SAMHD1, which depletes the cellular dNTPs required for viral DNA synthesis [7–19].

Tight regulation of the cellular dNTP concentration, and of the balance among the individual dNTPs (dATP, dGTP, dCTP and TTP), is required to prevent genomic instability and tumourigenesis [20,21]. SAMHD1 mutations have been shown to alter cellular dNTP concentrations [22,23] and are associated with the development of certain cancers [23–29] (reviewed comprehensively by Mauney and Hollis [30]). Furthermore, SAMHD1 catalytic activity modulates the efficacy of anti-cancer and anti-viral nucleoside analogues [31–40].

In addition to its dNTP triphosphohydrolase activity, it was reported that SAMHD1 possesses nuclease activity against DNA and RNA substrates [41–45]. However, the observed nuclease activity could not be replicated in other studies [46–48] and has been associated with a contaminant co-purified with recombinantly expressed SAMHD1 [46]. Despite lacking intrinsic nuclease activity, SAMHD1 binds nucleic acids [41,46,48–51], with selectivity for single-stranded over double-stranded oligonucleotides [46,50], and for RNA over DNA [46,49].

SAMHD1 may utilise dNTP hydrolase activity, nucleic acid-binding or a scaffolding activity in other cellular roles such as in DNA replication [52], DNA repair [53] and inhibiting LINE-1 retrotransposition [54–57]. Germline mutation to SAMHD1 or any of six other genes (TREX1, RNaseH2A, RNaseH2B and RNaseH2C, ADAR1 and IFIH1) can cause the auto-inflammatory condition Aicardi–Goutières Syndrome [30,58–65]. More broadly, SAMHD1 appears to repress immune responses to viral infection and other inflammatory stimuli [52,60,66–68]. However, these mechanisms remain to be elucidated.

## Regulation of cellular dNTP levels

The dNTP hydrolase activity of SAMHD1 is essential for both cellular dNTP homeostasis and regulating cell proliferation [24,29,69]. While SAMHD1 catalyses dNTP hydrolysis to reduce the cellular dNTP pool, several enzymes act antagonistically to increase the dNTP pool by catalysing dNTP synthesis either *de novo* or *via* salvage pathways. dNTPs are continually synthesised and degraded throughout the cell cycle, with the highest rates of dNTP flux occurring in S-phase [70]. dNTP levels in mammalian cells are ∼10- to 18-fold higher in S-phase than G_0_/G_1_ [70–72], and dNTP synthesis must continue during S-phase to complete chromosomal replication [72–75].

The catalytic activity of SAMHD1 is tightly controlled throughout the cell cycle through a mechanism of phosphorylation and dephosphorylation [3]. In S-phase, SAMHD1 appears to be phosphorylated at residue Thr592 by cyclin-dependent kinase 1 or 2 and cyclin A (CDK1/2-cyclinA) to lower the rate of dNTP hydrolysis [76–79]. At the end of M-phase, SAMHD1 catalytic activity is recovered due to dephosphorylation by phosphatase PP2A-B55α [80]. SAMHD1 expression levels may also vary throughout the cell cycle to further regulate dNTP hydrolase catalytic activity [3,4,30].

## Allostery

In addition to post-translational regulation, SAMHD1 is subject to allosteric regulation by nucleotides to fine-tune its catalytic activity. Allosteric regulation occurs when the binding of a ligand at one site affects the affinity of ligand binding or catalysis at a second site in the same protein. Allosteric effects can be positive, termed ‘allosteric activation’, or negative, ‘allosteric inhibition’. Allostery is observed in many proteins, from single-domains to large multimeric complexes [81–84]. SAMHD1 is allosterically activated by nucleotide binding in two allosteric sites, AL1 and AL2 [5,6,85–88]. However, there is little evidence to suggest allosteric site coordination modifies catalytic site selectivity in SAMHD1 [87]. This review focuses on the mechanism by which allostery regulates human SAMHD1 catalysis and so the experiments described refer to human SAMHD1 studies, unless explicitly stated otherwise.

## SAMHD1 domain organisation

Human SAMHD1 is a 626-residue protein that contains an N-terminal nuclear localisation signal, ^11^KRPR^14^ [89], a sterile-α-motif (SAM) domain (residues 34–113) and an HD catalytic domain (residues 115–599; Figure 1B–D). The SAM domain is an α-helical domain with an unknown function in SAMHD1 [90,91]. The HD catalytic domain is a phosphohydrolase domain that is named after the two pairs of Histidine–Aspartate residues (His167, His206, Asp207 and Asp311) that co-ordinate a metal ion at the catalytic site (Figure 1D) [92]. The HD domain of SAMHD1 is itself sufficient to catalyse the hydrolysis of dNTPs into 2′-deoxynucleoside and triphosphate [3,5,6,51,85,86,93].

Structural studies on human SAMHD1 have primarily focussed on the HD catalytic domain, as the flexibility of the linker connecting the SAM and HD domains has hindered structural studies on full-length human SAMHD1. More recently, X-ray crystal structures have been determined for mouse SAMHD1 containing both SAM and HD domains [94], providing some insight into how the human SAM and HD domains may interact with one another.

## Nucleotide-dependent tetramer assembly

**Figure 2.**
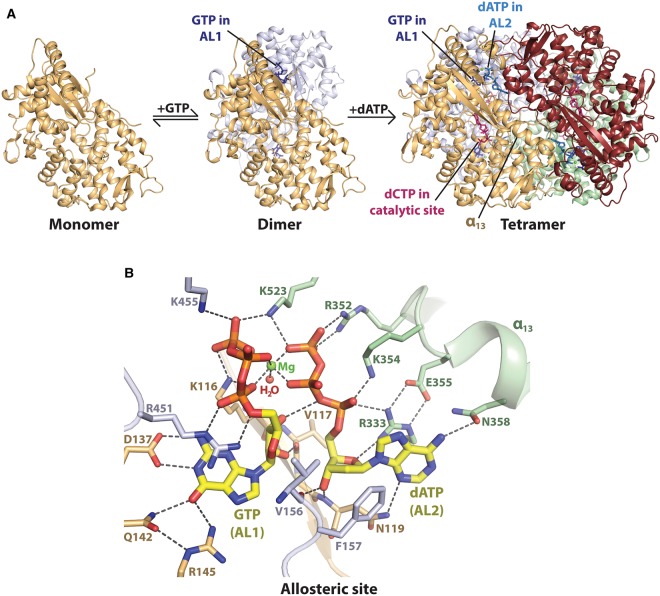
Nucleotide-dependent tetramerization of SAMHD1 HD domain. (**A**) Ordered pathway for tetramer assembly, proposed by Hansen et al. [97], and elucidated structurally using X-ray crystallography [77,78,85–88,94,95,101]. The four protein monomers of SAMHD1 are shown in light orange, grey, maroon and light green. From left to right: The apo SAMHD1 HD domain is in a monomer–dimer equilibrium with GTP (dark blue), which stabilises dimerisation by coordinating AL1 (PDB code: 4RXO) [95]. dATP (light blue) co-ordinates AL2, and a magnesium ion (green sphere) bridges adjacent AL1–AL2 allosteric sites to stabilise the HD domain tetramer (PDB code: 4RXP) [95]. dCTP in the catalytic site is shown in pink. (**B**) GTP–dATP coordination at adjacent allosteric sites, AL1–AL2, of an HD domain tetramer (PDB code: 4TO0) [87]. GTP in AL1 and dATP in AL2 are shown in stick representation, with C_α_ atoms shown in yellow. Hydrogen-bonding interactions are represented by dashed lines.

SAMHD1 is catalytically active as a homotetramer [93]. The HD domain tetramerises in a nucleotide-dependent manner (Figure 2A), with crystal structures revealing how nucleotides bind at two allosteric sites, AL1 and AL2, per protein monomer (Figure 2B) [85–88,95]. AL1 is specific for guanine-based nucleotides [85,87,96], which stabilise HD domain dimerisation [97]. The second allosteric site, AL2, lies adjacently to AL1 and is specific for a dNTP, which supports the dimer–dimer interactions required for tetramerization [85–88,95,97]. A magnesium ion co-ordinates the phosphoester oxygens of the nucleotides in AL1 and AL2 to further stabilise the tetramer [85], which explains the observation that SAMHD1 catalytic activity is magnesium-dependent [6]. Overall, four pairs of AL1–AL2 activators, each bridged by a magnesium ion, stabilise the SAMHD1 tetramer.

GTP is the likely physiological activator of AL1 [87,97–99], as the cellular concentration of GTP is greater than that of the other guanine-based nucleotides [100], and, unlike dGTP, GTP is not a substrate of SAMHD1 [5]. In AL1, the guanine base of a co-ordinated nucleotide is recognised through a hydrogen-bonding network involving residues Asp137, Gln142 and Arg145 [85,87,95]. AL1 has a preference for GTP ≥ dGTP > ddGTP [77,97,98], due to hydrogen bonds formed between the GTP ribose and the backbone carbonyl of Val117 and the adjacent AL2-co-ordinated dNTP [87,88,95]. The preference in AL1 for GTP > GDP >> GMP [98] is due to extensive salt-bridges formed between the GTP triphosphate, Lys116 of one SAMHD1 monomer, and Arg451 and Lys455 of a second monomer [85,86], explaining how AL1-coordination stabilises dimerisation.

In AL2, the bulky side chains of Val156 and Phe157 prevent binding by nucleotides functionalised at the 2′-ribose position, such as ribonucleoside-5′-triphosphates (NTPs), although fluoro-substitution at the 2′-proS position is tolerated [38,87,101]. AL2 selects for dNTPs over 2′,3′-dideoxynucleoside triphosphates (ddNTPs) due to two hydrogen bonds formed between the 3′-hydroxyl of a dNTP and the protein backbone of Asn119 and Val156 [85,86]. Salt-bridges between the dNTP triphosphate and residues Arg333, Arg352 and Lys354 further stabilise nucleotide coordination in AL2. Additionally, Lys523 forms salt-bridges with the γ-phosphates of each AL1–AL2 pair to select for triphosphorylated nucleotides in AL1 and AL2.

All four canonical dNTPs can co-ordinate AL2, with a preference for dATP > dGTP > TTP > dCTP [6,87,88]. The polar side chains of residues Asn119 and Asn358 and several water molecules adapt their hydrogen-bonding network to accommodate all four bases in AL2 [87]. The preference in AL2 for the purine nucleotides dATP and dGTP, over the pyrimidines TTP and dCTP, is due to more extensive cation-π stacking of the larger purine bases with the guanidino side chain of Arg333 [87]. Ji et al. [87] proposed that dATP is the primary activator of AL2, as they observed a stronger salt-bridge formed between the side chains of Arg333 and Glu355 only when dATP is bound in AL2. In contrast, dCTP is a poor AL2-activator of SAMHD1 [88], likely due to the inability of the cytosine base to form a direct hydrogen bond with Asn358.

## SAMHD1 tetramerization is essential for catalysis

Extensive experimental evidence supports the hypothesis that SAMHD1 tetramerization is essential for catalysing dNTP hydrolysis [85,86,88,93,97]. Firstly, dGTP, which can co-ordinate AL1, AL2 and the catalytic site, is hydrolysed by SAMHD1 *in vitro* in the absence of additional nucleotides [5,6,86,93], whereas the three other canonical dNTPs (dATP, dCTP and TTP) are only hydrolysed by SAMHD1 in the presence of AL1-activating GTP or dGTP [5,6,98]. Secondly, point mutations to key residues in AL1 (D137A, Q142A, R145A and R451E), AL2 (R333E) and the dimer–dimer interface (D361K, H364K and R372D) reduced SAMHD1 tetramerization and dNTP hydrolysis *in vitro* [77,85,86,88,93,95,102]. Finally, N- and C-terminal truncations in constructs 120–626 (Δ115–119) and 115–583 (Δ584–626), respectively, abolished tetramerization and reduced catalytic activity in comparison with constructs 1–626 and 115–626 [5,51,77], demonstrating the importance of tetramerization for catalysis.

## Long-lived, activated state of SAMHD1 corresponds to the homotetramer

Hansen et al. [97] observed that GTP and dNTPs, or dGTP alone, generate a long-lived, activated state of SAMHD1 that corresponds to the SAMHD1 homotetramer. Furthermore, the activated, homotetrameric state of SAMHD1 is not in equilibrium with free GTP or dNTP activators in solution. Strikingly, the SAMHD1 homotetramer persisted *in vitro* for hours without further exchange of nucleotides in AL1 or AL2 [77,97]. It is proposed that this slow rate of tetramer dissociation, despite activator depletion, enables SAMHD1 to deplete cellular dNTP concentrations to the nanomolar concentrations observed in macrophages and resting CD4^+^ T-cells.

## Thr592 phosphorylation destabilises the tetramer

Phosphorylation of human SAMHD1 residue Thr592 by CDK1/2-cyclinA regulates catalytic activity throughout the cell cycle [76–78]. Phosphorylation of residue Thr592 *in vitro* or introducing the phosphomimetic mutation T592E reduced tetramerization and catalytic activity [77,78]. The mutation T592E also eliminated the ability of SAMHD1 to restrict HIV-1 infection in macrophage-like PMA-differentiated U937 cells [77]. Similarly, another phosphomimetic mutation, T592D, also impaired the ability of SAMHD1 to block the lytic replication of the Epstein–Barr herpesvirus in producer Akata cells [103].

**Figure 3.**
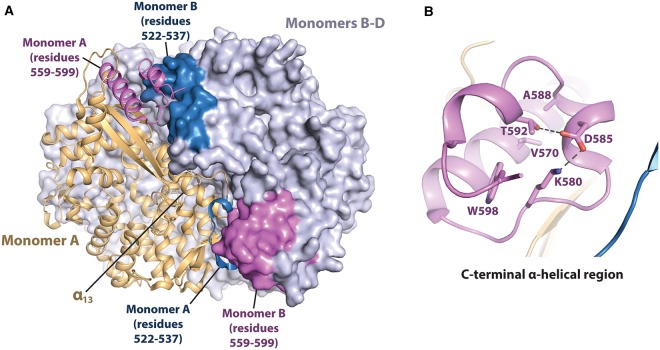
Thr592 phosphorylation modulates SAMHD1 tetramer stability. (**A**) X-ray crystal structure of SAMHD1 HD domain tetramer (PDB code: 4BZC) [85]. Monomer A is shown in cartoon representation in light orange. Monomers B–D are shown in surface representation in grey. Residues 559–599 are in pink and residues 522–537 are in blue. (**B**) C-terminal, the α-helical region between residues 559–599, comprising phosphorylated residue Thr592.

SAMHD1 residue Thr592 is buried in an α-helical region spanning residues 559–599, and this region interacts with residues 522–537 of an adjacent monomer at the dimer–dimer interface in the SAMHD1 tetramer (Figure 3A). From crystal structures, it appears that phospho-T592 would experience electrostatic repulsion with the adjacent acidic residue Asp585 and may sterically clash with hydrophobic residues Val570 and Trp598 (Figure 3B) [77,78]. Correspondingly, crystal structures of phospho-T592 or T592E SAMHD1 reveal that phosphorylating Thr592 or making the phosphomimetic mutation T592E disrupts local protein folding, causing the C-terminal residues 585–599 to become disordered in the crystal lattice [77,78].

Molecular dynamics (MD) simulations by Patra et al. [104] showed that mutation T592E caused minor local perturbations to residues 585–595, but did not affect the integrity of the allosteric or catalytic sites on the timescale modelled. Further analysis of correlated motions across the SAMHD1 tetramer in the MD simulations revealed that the mutation T592E decoupled a signalling pathway between residue Thr592 and the allosteric sites, and increased the dynamic coupling between Thr592 and α-helix 13 (α_13_; residues 352–375) at the dimer–dimer interface [105]. The authors concluded that phosphorylation of Thr592 may trigger a loosening of the HD domain tetramer.

## Catalytic site selectivity

**Figure 4.**
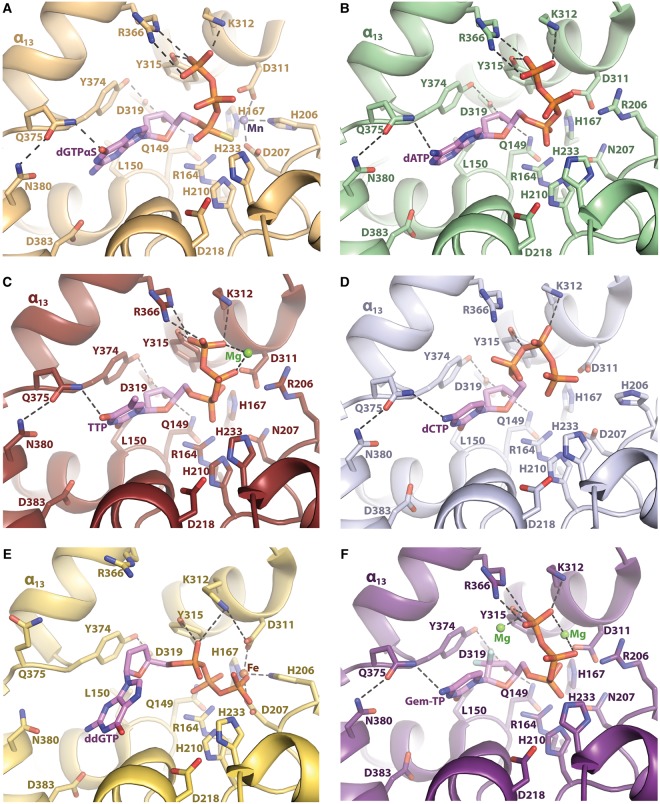
SAMHD1 catalytic site accommodates dNTPs and dNTP analogues. (**A**–**F**) Nucleotide coordination in the catalytic site of the human SAMHD1 HD domain. (**A**) WT SAMHD1 with dGTPαS (PDB: 4BZC) [85]. (**B**) H206R/D207N SAMHD1 with dATP (PDB: 4QG1) [88]. (**C**) H206R/D207N SAMHD1 with TTP (PDB: 4TNZ) [87]. (**D**) WT SAMHD1 with dCTP (PDB: 4RXR) [95]. (**E**) WT SAMHD1 with ddGTP (PDB: 5AO1) [77]. (**F**) H206R/D207N SAMHD1 with gemcitabine-TP (Gem-TP) (PDB: 6DW5) [101]. Nucleotides are shown in stick representation with C_α_ atoms in pink. Manganese (purple), magnesium (green) and iron (brown) metal ions are represented as spheres.

The SAMHD1 catalytic site can accommodate all four canonical dNTP substrates (dATP, dGTP, dCTP and TTP), as well as dUTP and a variety of dNTP analogues (Figure 4). In each case, SAMHD1 catalyses their hydrolysis into triphosphate and 2′-deoxynucleoside products. As all four canonical dNTPs can be accommodated in the catalytic site, they also act as competitive inhibitors of one another's hydrolysis in mixed dNTP pools [87,97,98]. Chromatography-based dNTP hydrolysis experiments, which were performed in the presence of GTP and all four dNTPs to minimise allosteric effects on catalysis, demonstrated the following rank order for dNTP hydrolysis rate: dGTP > dCTP > TTP > dATP [5,6,87]. Co-crystallization experiments by Ji et al. [87] supported a catalytic site-binding preference of: dCTP > dGTP ≈ TTP > dATP.

Base selectivity in the catalytic site is achieved through subtle differences in the hydrogen bonding between a dNTP base, its network of hydrating water molecules and residues Leu150, Tyr374, Gln375, Asn380 and Asp383, which line the catalytic pocket [87,88]. The side chains of residues Leu150, Tyr315 and Tyr374 form a tight-binding pocket around the base and 2′-deoxyribose moieties of a dNTP substrate [38,85,101]. The 3′-hydroxyl group on a dNTP substrate is hydrogen bonded by the polar side chains of residues Gln149 and Asp319 [86,88]. Nucleotide binding in the catalytic site is further stabilised by salt-bridges between the triphosphate and the basic side chains of Arg164, Lys312 and Arg366 [85,88].

In addition to canonical dNTPs and dUTP, the SAMHD1 catalytic site can co-ordinate and hydrolyse particular dNTP analogues. The poorly hydrolysed analogue ddGTP co-ordinates the SAMHD1 catalytic site, as revealed through crystal structures (Figure 4E) [77]. Knecht et al. [101] solved co-crystal structures in which the catalytic site was occupied by the triphosphorylated forms of anti-cancer drugs cladribine, clofarabine, fludarabine, cytarabine and gemcitabine (Figure 4F), and the anti-viral agent vidarabine. The authors’ structural and biophysical studies revealed that the catalytic site could tolerate fluoro- and chloro-substitutions at the carbon-2 position on an adenine base, fluoro- and hydroxyl-substitutions at the 2′-proS ribose position, and a fluoro-group at the 2′-proR position. Such substitutions at the 2′-proS position are tolerated by a compensatory rotation of the ribose moiety of these analogues within the catalytic pocket. Leu150 and Tyr374 side chains prevent bulkier functionalisation at the 2′-proR position, while Tyr315 prevents functionalisation to the 3′-proS position [38,85,101].

## dNTP geometry in the catalytic site

Numerous crystal structures have been solved of the SAMHD1 HD domain with dNTPs or dNTP analogues in the catalytic site [77,78,85–88,94,95,101]. Frequently, the inactivating double mutation H206R/D207N has been employed in these studies [78,85,87,88,101]. The H206R/D207N mutation to the HD motif (His167, His206, Asp207 and Asp311) prevents coordination of a metal ion at the HD motif and eliminates catalytic activity in SAMHD1 [85,93]. Metal ion coordination at the HD motif is likely important for catalysis, as a further HD motif mutant, D311A, is also catalytically inactive [5,44,106].

While it is possible that dNTP or dNTP analogue coordination may be perturbed in crystal structures of SAMHD1 mutant H206R/D207N (Figure 4B,C,F), a similar binding mode is observed for the analogue dGTPαS in a wild-type (WT) catalytic site (Figure 4A) [85]. The consensus between independently reported H206R/D207N-dNTP and WT-dGTPαS structures (Figure 4A–C) [85,87,88] suggests there may be a physiological basis for this nucleotide-binding mode in the catalytic site. Therefore, it could be postulated that these non-catalytically competent SAMHD1-nucleotide structures represent enzyme–substrate complexes prior to catalysis.

In comparison, a different triphosphate geometry is modelled in the crystal structures of catalytically competent WT–dNTP complexes (Figure 4D) [86,95]. The base and 2′-deoxyribose portions of the dNTP ligands superimpose with those of non-catalytically competent H206R/D207N-dNTP structures. However, the triphosphate moiety is modelled in different configurations. Thus, the WT–dNTP structures may represent intermediate- or product-like states during catalysis.

Structures of WT SAMHD1 with the poorly hydrolysed analogue ddGTP reveal a further binding mode for the nucleotide in the catalytic site [77], with ddGTP less well buried within the catalytic pocket (Figure 4E). The WT–ddGTP crystal structures reveal a unique substrate-binding mode that may be required for ddGTP hydrolysis, but importantly could represent a nucleotide-bound state along the dNTP substrate-binding pathway of SAMHD1. Further SAMHD1-nucleotide structural studies may be required to elucidate nucleotide-binding modes at various stages of catalysis, including substrate binding, hydrolysis and product release.

## Catalytic mechanism

The chemical reaction catalysed by SAMHD1 was initially identified through chromatography-based experiments in which dNTP substrates were demonstrated to be hydrolysed directly into 2′-deoxynucleoside and triphosphate products (Figure 1A), rather than by sequential monophosphate cleavages *via* 2′-deoxynucleoside-5′-diphosphate (dNDP) and 2′-deoxynucleoside-5′-monophosphate (dNMP) intermediates [5,6]. The catalytic mechanism was further investigated using mass spectrometry experiments that determined oxygen from bulk water is incorporated into the triphosphate product, rather than the 2′-deoxynucleoside product, supporting a mechanism of nucleophilic attack on the α-phosphorous that results in cleavage of the α-phosphorous-to-5′-oxygen covalent bond [107].

In addition to residues in the HD motif, residues His210, Asp218 and His233 have been proposed to be important for catalysis, based on the observation that mutations H210A and H233A disrupt catalysis [88], and on the conservation of these three residues across HD phosphohydrolase domains, including in the homologous protein EF1143 from the bacterium *Enterococcus faecalis* [6,85,108]. Furthermore, a crystal structure of mutant H210A was found to lack nucleotide coordination in the catalytic site, supporting a function for residue His210 in substrate dNTP coordination [88].

## ‘Open’ and ‘closed’ HD domain conformations

**Figure 5.**
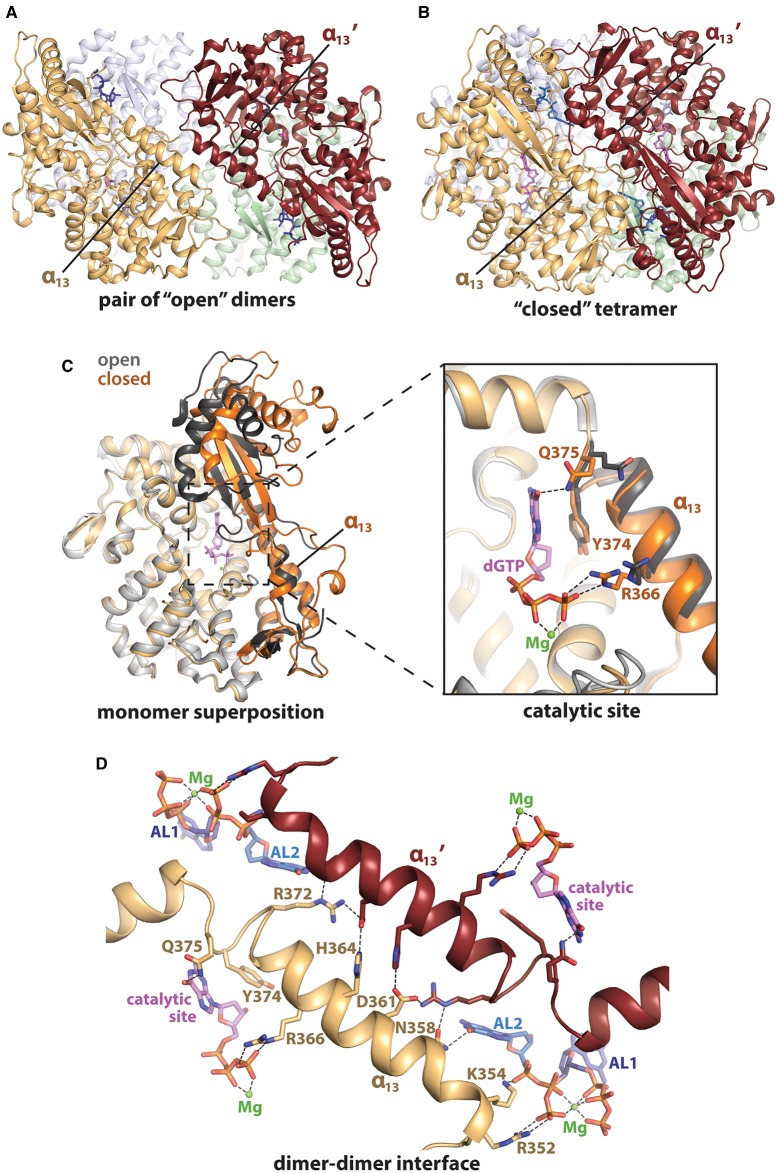
‘Open’ and ‘closed’ conformations of HD domain. (**A**) X-ray crystal structure of the HD domain in ‘open’ conformation co-ordinated to GTP (dark blue) in AL1 (PDB: 4RXO) [95]. The tetramer comprises two dimers, with one formed from the orange and grey monomers, and the second from the maroon and light green monomers. (**B**) X-ray crystal structure of HD domain in ‘closed’ tetrameric conformation, with dGTP co-ordinated in AL1, AL2 and the catalytic site (PDB: 4BZB) [85]. dGTP is shown in dark blue (AL1), pale blue (AL2) or pink (catalytic site) and magnesium ions are shown as green spheres. (**C**) Superposition of an HD domain monomer in ‘open’ (grey) and ‘closed’ (orange) conformations. Residues 326–375 and 454–599 differ in their conformation between the ‘open’ and ‘closed’ states and are represented by darker shades of grey and orange, respectively. The dGTP- and magnesium-coordination observed in the catalytic site of the ‘closed’ state is displayed, while the manganese- and phosphate-coordination observed in the ‘open’ state is not shown. (**D**) Interactions formed by the α_13_ helix at the dimer–dimer interface (residues Asn358, Asp361, His364 and Arg372), in the catalytic site (Arg366, Tyr374 and Gln375) and in AL2 (Arg352, Lys354 and Asn358). One monomer is shown in pale orange, and its neighbouring monomer at the dimer–dimer interface is shown in maroon. dGTP in AL1 (dark blue), AL2 (pale blue) and the catalytic site (pink) is shown in stick representation. Magnesium ions are represented by green spheres. Hydrogen bonds are shown as dashed lines.

Crystal structures of human SAMHD1, either apo or with nucleotides co-ordinated, reveal that the HD domain contains intrinsic conformational flexibility [5,77,85,86,95,102], adopting two distinct conformations in crystal structures, which we term the ‘open’ and ‘closed’ conformations (Figure 5A–C). In the absence of co-ordinated nucleotides, or with only GTP bound in AL1, the SAMHD1 HD domain adopts an ‘open’ conformation (Figure 5A), with a more expanded catalytic site pocket, and is disordered between residues 278–283, 507–546 and 583–599 [5,77,95,102]. In the crystal lattices of ‘open’ structures, the HD domains are arranged in dimeric repeating units. These dimeric units likely correspond to the SAMHD1 dimer of the monomer–dimer equilibrium that is present in solution in the absence of nucleotides or in the presence of only GTP [93,97].

SAMHD1 HD domain crystal structures with nucleotides simultaneously bound in AL1, AL2 and the catalytic site adopt a so-called ‘closed’ conformation (Figure 5B) that is more compact about the catalytic site, and ordered to a greater extent, with density observed for the HD domain backbone for all residues between positions 115–599, except for a short loop between residues 278–283 [85]. In ‘closed’ structures, the HD domains assemble in the crystal lattice into homotetramers that contain D2 dihedral symmetry, whereby the four monomers are related to one another by three 2-fold symmetry axes.

Structural comparisons suggest that the HD domain must undergo a change in conformation during the dimer-to-tetramer transition to accommodate dNTPs into AL2 and the catalytic site. Secondary structural elements are conserved between dimeric (‘open’) and tetrameric (‘closed’) conformations of the SAMHD1 HD domain. However, between dimeric and tetrameric structures, motions of up to ∼5 Å affect tertiary packing in two regions of the protein, between residues 326–375, and 454–599 (Figure 5C) [85,86]. Several residues in these two regions are important for nucleotide coordination in AL1, AL2 and the catalytic site. Therefore, it is likely that these regions have important functions in allosteric regulation in SAMHD1.

## Linkage between allosteric and catalytic sites

As described above, the HD domain conformation varies between dimeric (apo or AL1-occupied) and tetrameric (AL1-, AL2- and catalytic site-occupied) states of SAMHD1. While the majority of residues across the catalytic site do not appear to be significantly structurally perturbed during tetramer assembly, tertiary structural changes alter the positioning of catalytic site residues Arg366 and Gln375 (Figure 5C), which lie on one face of α_13_ [85,86]. Residues Arg366 and Gln375 are involved in dNTP coordination in the catalytic site of closed, tetrameric SAMHD1 structures, but appear too distal for substrate coordination in open, dimeric structures that lack dNTPs in AL2 and the catalytic site.

Helix α_13_, which spans residues 352–375, bridges the catalytic and allosteric sites, and makes important interactions at the dimer–dimer interface (Figure 5D) [86]. Catalytic site residues Arg366 and Gln375 are at the C-terminal end of α_13_, while residues Arg352, Lys354 and Asn358 at the N-terminal end of α_13_ are involved in dNTP coordination in AL2. At the dimer–dimer interface, the α_13_ helix of one monomer interacts with the neighbouring monomer's helix, α_13_', through a network of salt-bridges and hydrogen bonds involving residues Asn358, Asp361, His364 and Arg372 from both α_13_ and α_13_' elements. Thus, α_13_ appears to be crucial for allosteric regulation, by communicating allosteric site occupancy and tetramerization to the catalytic site, with residues Arg366 and Gln375 supporting substrate dNTP binding once AL2 is occupied and the protein has tetramerized.

In addition to structural changes in the catalytic site, HD domain tetramerization likely alters protein dynamics. Patra et al. [104,105] explored mechanisms for cross-talk between allosteric and catalytic sites in SAMHD1 using correlation analysis of MD simulations. The authors observed that correlated motions between allosteric and catalytic sites were reciprocated across the HD domain tetramer, revealing both short-range and long-range allosteric signal transduction in SAMHD1. Furthermore, removing dATP from one AL2 site in the SAMHD1 HD domain tetramer significantly reduced the rigidity of the protein around the dATP-occupied catalytic site. In separate MD simulations, Cardamone et al. [109] observed that removing all nucleotides and magnesium ions from the protein tetramer weakened α_13_–α_13_' interactions at the dimer–dimer interface, and the AL1 mutation R145E accelerated the destabilisation of the tetramer [110]. Overall, biophysical and computational experiments demonstrate that interactions at the dimer–dimer interface and nucleotide occupancy at the allosteric sites modulate the catalytic site structure and dynamics in order to regulate catalysis.

## Summary

Allosteric site occupancy and HD domain tetramerization control both the structural integrity and the rigidity of the SAMHD1 catalytic site [85,86,105,109,110]. In the absence of nucleotide coordination in AL1, AL2 and the catalytic site, SAMHD1 exists in a monomer–dimer equilibrium [93,97]. GTP or dGTP binding in AL1 increases the proportion of dimeric SAMHD1 [97] and is necessary for subsequent dNTP binding in AL2 [97]. Changes in tertiary structure and protein dynamics result from the dNTP-induced dimer-to-tetramer transition, including structural perturbations to residues 326–375 and 454–599 [85,86], and changes in the dynamics of catalytic site residues, including His206, Tyr374 and Gln375 [104]. The catalytic site becomes more rigid upon nucleotide-induced tetramerization [104,105], and there appears to be an energetic coupling between nucleotide binding in AL2 and the catalytic site [97]. Subsequent to catalysis, the reaction products, 2′-deoxynucleoside and triphosphate, dissociate from SAMHD1 and the catalytic site of a closed tetramer appears sufficiently accessible for nucleotide exchange to occur without tetramer disassembly. Kinetic experiments demonstrate that the AL1- and AL2-co-ordinated tetramer is a long-lived, activated state, in which the AL1- and AL2-co-ordinated nucleotides are not in exchange with free nucleotides [97]. This is relevant to a cellular environment in which the dNTP pool has been largely depleted. Stable, active SAMHD1 tetramers persist [77,97] and hydrolyse dNTPs to drive the cellular dNTP pool to nanomolar concentrations that are observed in resting cells and are required for the restriction of HIV-1 replication.

**Perspectives**

- SAMHD1 has important anti-viral, anti-cancer and anti-inflammation functions in the cell. SAMHD1 restricts HIV-1 replication in dendritic and myeloid lineage cells. Mutations to SAMHD1 have been identified in hypermutated cancers, and germline mutation to SAMHD1 can cause Chronic Lymphocytic Leukaemia and auto-immune condition Aicardi–Goutières Syndrome.
- The dNTP triphosphohydrolase catalytic function of SAMHD1 is essential for HIV-1 restriction but also for cellular dNTP homeostasis. The catalytic domain of SAMHD1 tetramerizes in a nucleotide-dependent manner, with GTP and dATP coordinating two allosteric sites (AL1 and AL2) per SAMHD1 monomer to stimulate dNTP hydrolysis in the catalytic site. This review combines the results of biophysical, structural and MD studies to present a unified mechanism for the allosteric regulation of catalysis by SAMHD1.
- X-ray crystallographic studies have revealed how nucleotide coordination in AL1 and AL2 stabilises catalytic domain tetramerization. However, it remains unclear how a substrate dNTP is co-ordinated in the WT catalytic site of SAMHD1 prior to catalysis and how SAMHD1 catalyses dNTP triphosphohydrolysis. Further studies are required to elucidate the catalytic mechanism of SAMHD1.

## Abbreviations

AL1: allosteric site 1 of SAMHD1
AL2: allosteric site 2 of SAMHD1
CDK1/2-cyclinA: cyclin-dependent kinase 1 or 2 and cyclin A
ddNTP: 2′,3′-dideoxynucleoside-5′-triphosphate
dNDP: 2′-deoxynucleoside-5′-diphosphate
dNMP: 2′-deoxynucleoside-5′-monophosphate
dNTP: 2′-deoxynucleoside-5′-triphosphate
Gem-TP: gemcitabine-triphosphate
HD: Histidine–Aspartate motif or domain
MD: molecular dynamics
NTP: ribonucleoside-5′-triphosphate
PDB: Protein Data Bank
SAM: sterile-α-motif domain
SAMHD1: sterile-α-motif and HD domain containing protein 1
WT: wild-type
α_13_: α-helix 13
